# Analysis of resorbable mesh implants in short-term human muscular fascia cultures: a pilot study

**DOI:** 10.1007/s10029-020-02271-x

**Published:** 2020-07-28

**Authors:** V. Trapani, G. Bagni, M. Piccoli, I. Roli, F. Di Patti, A. Arcangeli

**Affiliations:** 1 Department of General and Emergency Surgery, New Sant’Agostino-Estense Hospital NOCSAE, via Pietro Giardini 1355, Baggiovara, Modena, Italy; 2 Department of Experimental and Clinical Medicine, Section of Internal Medicine, University of Florence, viale GB Morgagni 50, 50134 Florence, Italy; 3 Department of Physics, University of Florence, Sesto Fiorentino, Florence, Italy; 4 CSDC-Center for the Study of Complex Dynamics, Sesto Fiorentino, 50019 Florence, Italy

**Keywords:** Hernia, Ex vivo model, Collagen I, Collagen III, Polyhydroxybutyrate, Polypropylene

## Abstract

**Purpose:**

Alteration in fascial tissue collagen composition represents a key factor in hernia etiology and recurrence. Both resorbable and non-resorbable meshes for hernia repair are currently used in the surgical setting. However, no study has investigated so far the role of different implant materials on collagen deposition and tissue remodeling in human fascia. The aim of the present study was to develop a novel ex vivo model of human soft tissue repair mesh implant, and to test its suitability to investigate the effects of different materials on tissue remodeling and collagen composition.

**Methods:**

Resorbable poly-4-hydroxybutyrate and non-resorbable polypropylene mesh implants were embedded in human abdominal fascia samples, mimicking common surgical procedures. Calcein-AM/Propidium Iodide vital staining was used to assess tissue vitality. Tissue morphology was evaluated using Mallory trichrome and hematoxylin and eosin staining. Collagen type I and III expression was determined through immunostaining semi-quantification by color deconvolution. All analyses were performed after 54 days of culture.

**Results:**

The established ex vivo model showed good viability at 54 days of culture, confirming both culture method feasibility and implants biocompatibility. Both mesh implants induced a disorganization of collagen fibers pattern. A statistically significantly higher collagen I/III ratio was detected in fascial tissue samples cultured with resorbable implants compared to either non-resorbable implants or meshes-free controls.

**Conclusion:**

We developed a novel ex vivo model and provided evidence that resorbable polyhydroxybutyrate meshes display better biomechanical properties suitable for proper restoration in surgical hernia repair.

## Introduction

Reinforcement of the abdominal wall with mesh implants is the most widely applied and established procedure in abdominal hernia surgical repair procedures. Both non-resorbable and fully resorbable soft tissue repair implants are currently available. The latter accounts for the strength of a synthetic mesh, and shows the remodeling characteristics of a biologic graft. However, all meshes inevitably cause a foreign body reaction which affects collagen deposition, and in particular the type I/type III collagen ratio. This further alters connective tissue strength due to a delayed induction in collagen I conversion from immature collagen III [1, 2]. Changes in this ratio affects biomechanical strength and stability in already injured fascial tissue and may therefore increase the risk of hernia recurrence.

Collagen type I is indeed the characteristic component of mature scars or fascial tissues, while collagen type III represents the mechanically unstable, less cross-linked subtype, synthesized during early wound healing stages [3].

Several studies already showed that the unbalanced ratio between the two collagen types might represent one of the main pathogenetic aspects of hernias’ development, with the lower tensile strength of collagen type III being a major factor in disease development and recurrence risk [4–6]. A first study reported diminished collagen content in the sheath of the rectus abdominis muscles as a direct or indirect cause of inguinal hernias development [7]. Moreover, immunohistochemistry analysis revealed a decrease in collagen I/III ratio in the fascial tissues of patients with hernias compared to healthy controls [8, 9]. It has also been suggested that individuals with abnormalities in collagen production might have a higher chance of developing hernias [10]. Consistently, systemic increase in the synthesis of type III collagen may result in reduced collagen fibril assembly in the abdominal wall, eventually leading to herniation [11]. Altogether, these findings indicate the dependence of abdominal wall mechanical stability on collagen composition.

Although both preclinical and clinical studies showed that distribution and quantity of the synthesized collagen is directly influenced by the type of mesh implant, it is still not clear if the latter has any influence on collagen content composition.

To address this point, we developed a pilot study aimed to assess the feasibility of a novel ex vivo model of abdominal hernia mesh implant, to determine the effects of two different mesh implant materials (resorbable poly-4-hydroxybutyrate and non-resorbable polypropylene) on collagen production and fascial tissue remodeling in vitro. In particular, we developed and tested for the first time a novel tissue culture model, in which tissue samples from a small cohort of patients were cultured for 54 days in the presence of different mesh implant materials, and both vitality and collagen I vs collagen III deposition were evaluated.

## Samples and methods

### Fascial tissue samples

A total of 7 consecutive patients undergoing open abdominal procedures for other reasons were enrolled for the study at Sant’Agostino-Estense hospital in Baggiovara (Modena, Italy). Adult patients (older than 18 years) with indication to open surgery for both benign and oncological pathologies in elective setting were included; BMI > 30, a proven clinical immunodeficient status, emergency procedures, previous abdominal surgery and the presence of primary or ventral abdominal wall hernias represented criteria of exclusion.

In particular, at the time of the procedure, under general anesthesia a median incision was performed, subcutaneous tissue was dissected and aponeurosis was exposed; biopsy of surgical specimen was taken in correspondence of the section margin of posterior fascia on the midline without using the electrical cautering.

Considering the early explorative nature of this study, sample size was determined based on the aim to show preliminary methods feasibility and to obtain an estimate of effect size. No statistical power analysis was applied.

Subject demographics are summarized in Table 1.

**Table 1:** Subjects demographics

Subject ID	Sex (male/female)	Age (years)
GB 001	Male	85
GB 002	Male	72
GB 003	Female	81
GB 004	Male	70
GB 005	Male	72
GB 006	Female	74
GB 007	Female	80

### Mesh implants

Surgical mesh implants, intended for soft tissue repair, were developed by Bard (CR Bard Inc-BD Medical, USA).

Implants consisted in two different types of materials, one being a monofilament fully resorbable macroporous poly-4-hydroxybutyrate (P4HB) mesh (Phasix™) while the other a non-resorbable monofilament polypropylene mesh (Perfix™).

### Samples handling, ex vivo mesh implant and culture conditions

Fascia samples were shipped right after surgical explant in 45 ml RPMI medium supplemented with 10% FBS and 1% Penicillin–Streptomycin (EuroClone, Milan, Italy) at room temperature.

**Fig. 1:**
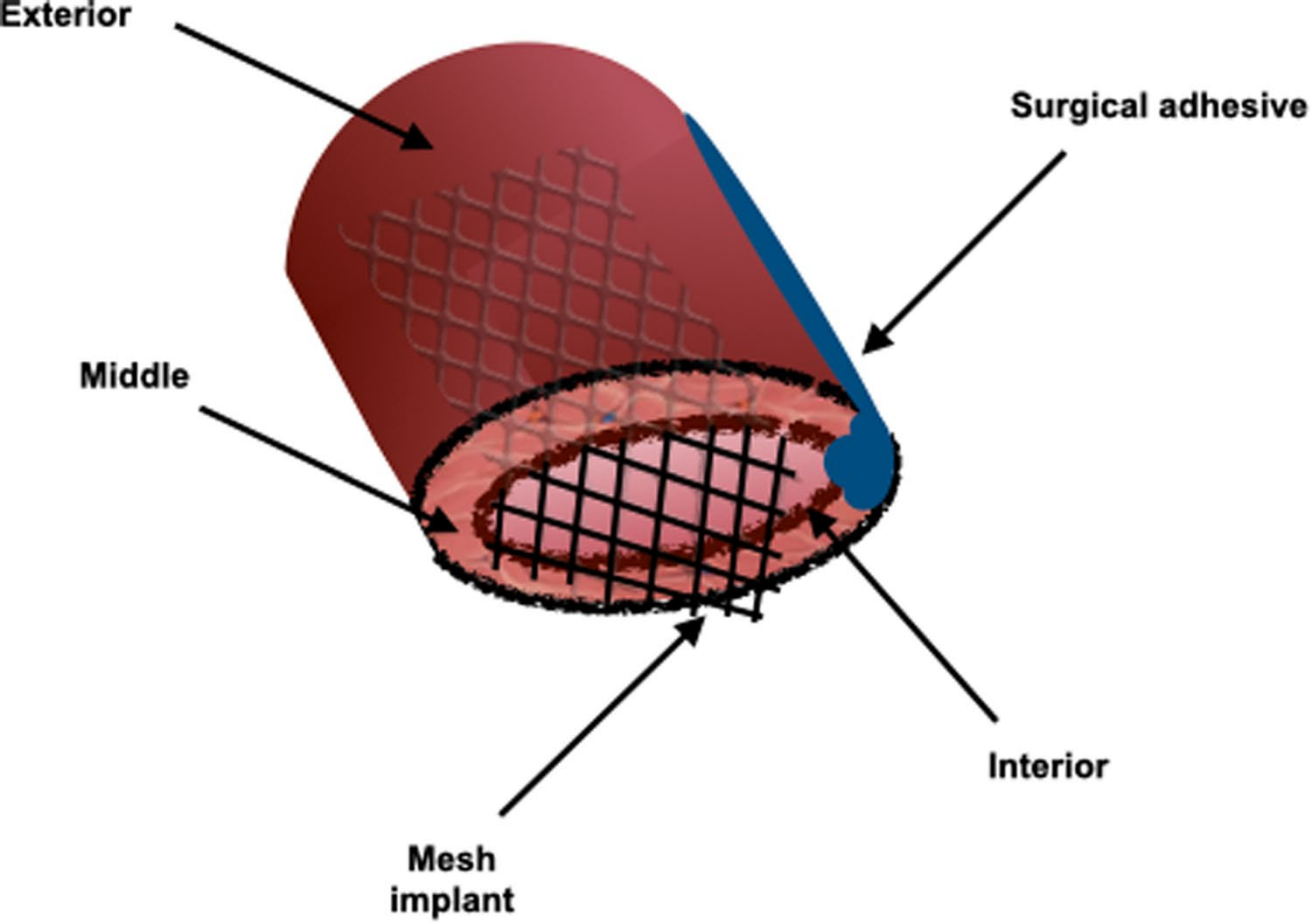
Fascia samples ex-vivo mesh implant model representation

Samples were immediately divided into three pieces (measuring approximately 0.6 × 0.6 cm) at the time of arrival using a scalpel, to be then processed as follows: (i) resorbable Phasix™ mesh implantation (R), (ii) non-resorbable Prefix™ mesh implantation (NR) or (iii) mesh-free controls (C). Briefly, small meshes cut-outs were put in contact with fascia samples in a “sandwich-like” configuration and secured using the smallest amount of cyanoacrylate-based tissue adhesive (PeriAcryl 90 HV, Gluestich Inc, Canada) as represented in Fig. 1. This type of adhesive allows proper mesh fixation, showing good tissue integration and effective short-term biocompatibility both in vivo and in vitro, and therefore represents a feasible alternative approach to fix mesh instead of using suture [12, 13]. Moreover, cyanoacrylate-based adhesive is also specifically used in the clinical setting as a common method for soft tissue repair mesh fixation and it was therefore selected as the most appropriate method for mesh fixation.

Samples which didn’t undergo mesh implant were considered as controls (C) and underwent the application of cyanoacrylate adhesive in order to achieve the same three-dimensional model conformation applied in the other study conditions.

All samples were kept fully submerged in 20 ml RPMI medium supplemented with 10% FBS (EuroClone), 1% Amphotericin-B (EuroClone, Italy) (2.5 mg/L) and 1% Penicillin–Streptomycin solution (EuroClone) at 37 °C and 5% CO_2_ in suspension culture flasks. Culture medium was replaced weekly with fresh one. All the above-mentioned procedures were performed under sterile conditions.

### Samples viability assessment

Samples viability was evaluated at the time of samples arrival (*T*_0_) and 54 days following meshes implant (54 days) using Calcein-AM/Propidium Iodide vital staining. Mesh-free samples (C) were also evaluated at the two overmentioned time points.

Calcein-AM selectively stains viable cells in bright green, while Propidium Iodide (PI) is used to identify dead cells as bright red.

Briefly, samples portions measuring approximately 0.20 × 0.20 cm, were obtained at each time point and for each condition using a scalpel under sterile conditions. They were then incubated in 2 μg/mL Calcein-AM (Molecular Probes, USA) and 10 μg/mL Propidium Iodide (Sigma-Aldrich, USA) in PBS at 37 °C for one hour in 24-wells culture plates (500 ml/well).

Tissue samples were then washed twice in PBS to remove excess staining.

Images were acquired using a fluorescence microscope system and the RS Image Software (Photometrics, USA). Calcein was detected at 450 nm/490 nm, while PI at 510 nm/530 nm.

Intensity of the two fluorescence channels was quantified separately as integrated density (IntDen) and subjected to background subtraction using ImageJ (National Institutes of Health-NIH, USA).

Fluorescence quantification was performed in three randomly selected 10X microscopic fields per sample, in each culture condition detailed above. In order to evaluate culture conditions effect on samples viability, results at 54 days were expressed as percentage of T_0_ control samples.

### Mallory trichrome staining

In order to evaluate total collagen composition of fascia samples, Mallory trichrome staining was performed. This staining allows the identification of collagen fibers in blue and muscle fibers in red.

Meshes implants were carefully removed from samples using a scalpel, taking care to avoid tissue damage. C, R and NR samples portions measuring approximately 0.2 × 0.2 cm, fixed in 4% formaldehyde for 12 h, were dehydrated and paraffin embedded. After sectioning at 7 μm, air-dried slides were de-paraffinized and rehydrated.

Sections were then placed into 1% acid fuchsin solution for 3 min, followed by 2 min in 1% phosphomolybdic acid and 15 min in Mallory staining solution (2% Orange G, 0.5% methyl blue and 2% oxalic acid in distilled water). Each staining step was followed by a quick rinse in distilled water. Section were then dehydrated and differentiated in ethanol, cleared with xylene and mounted using a resinous medium.

### Hematoxylin and eosin staining

Standard Hematoxylin and Eosin staining was performed on C, R and NR samples at *T*_0_ and 54 days in order to qualitatively evaluate tissue morphology. Samples were processed as previously described for Mallory staining and sectioned at 7 μm.

### Type I and type III collagen immunostaining

Type I and III collagen content was assessed in paraffin-embedded 7 μm samples slices using the HRP-DAB Ultravision LP Detection System kit (Thermo Scientific, USA) following manufacturer’s instruction.

A heat-induced microwave antigen retrieval protocol (4 heating steps at 700 W, 5 min each) in Sodium Citrate buffer (Ph 6.0) was performed before the immunostaining procedure.

Slides were incubated overnight at 4 °C with 50 μl of primary antibodies (rabbit polyclonal anti-human type I collagen-ab34710 or mouse monoclonal anti-human type III collagen-ab6310, both provided by Abcam) diluted 1:500 in Ultra V Block solution 1:10 in PBS.

Finally, all sections were counterstained with Mayer’s Hematoxylin, dehydrated in ethanol, cleared in xylene and mounted using a resinous medium.

Negative control sections were prepared by omitting the primary antibody in order to ensure staining specificity. In all cases, control sections showed no staining under microscopic examination.

Immunostained slides were visualized using a Leica DMR light microscope paired with a Leica DC 200 CCD camera system and DCViewer v3.2 software for white balance and image acquisition.

Identical reagents, processing, DAB incubation time and image acquisition settings were applied for all slides.

Images were then exported in ImageJ v1.51 (National Institutes of Health-NIH, USA) and background subtraction (rolling ball radius = 50.0 pixels) was performed.

Immunostaining intensity was quantified with Fiji software [14] using the built-in H DAB color deconvolution vector based on stain-specific red–green–blue (RGB) absorption to isolate the brown DAB from hematoxylin counterstain and unspecific background [15, 16].

Staining intensities were calculated as grey-scale values and converted in optical density (OD) using the formula log(Max intensity/Min intenisty).

For each subject, five randomly selected 40X whole-stained microscopic fields per sample condition were chosen and quantified.

### Data analysis

Data statistical and graphical analysis was carried out using Origin V.8 (OriginLab Corporation, USA) and GraphPad Prism 6 software (GraphPad Software, USA).

Results were expressed as mean ± SEM. Since data represent repeated measurements within each study condition and are normally distributed, statistical comparisons between multiple groups were performed using the “Repeated Measure ANOVA” (RA) test, followed by Tukey’s post-hoc test. For all tests, differences with *P* < 0.05 were considered statistically significant.

## Results

### Samples viability

**Fig. 2:**
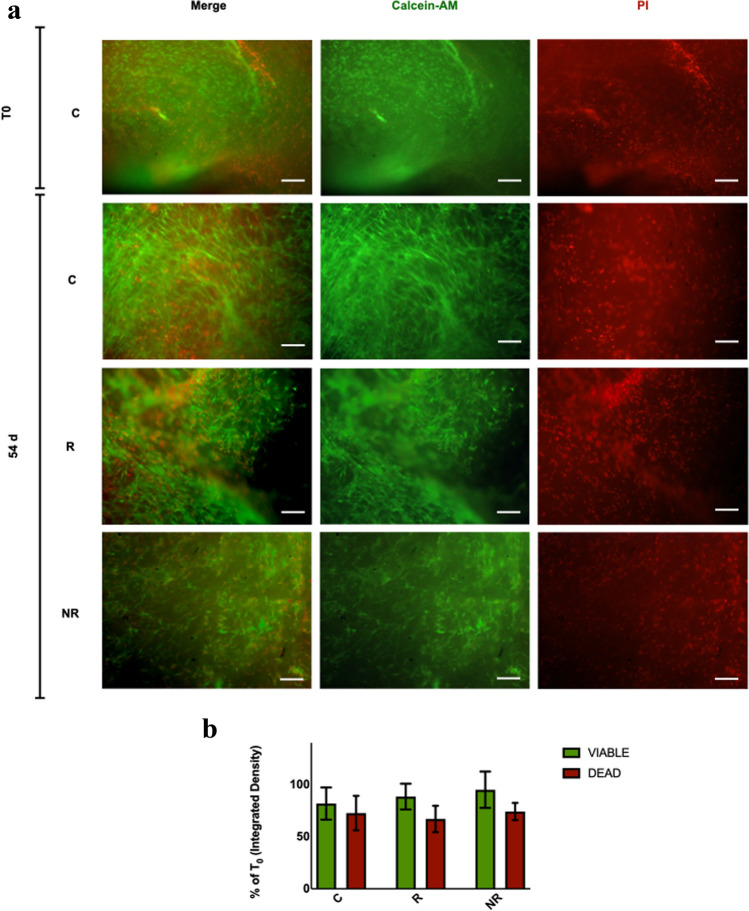
Samples viability assessment using Calcein-AM/Propidium Iodide vital staining. **a** Representative single-channel and merged images (10× magnification, scale bar = 100 µm); **b** Fluorescence intensity calculated as Integrated Density (IntDen) and presented as *T*_0_ control percentage. Bars are representative of the mean ± SEM of three randomly selected 10X microscopic fields per condition for each sample (*n* = 7). *C* samples which did not undergo mesh implant, mesh-free controls, *R* fully resorbable poly-4-hydroxy-butyrate mesh implant, *NR* non-resorbable polypropylene mesh implant, *T*_0_ time of tissue samples arrival, *54d* 54 days culture from meshes implantation

Tissue samples vital staining revealed the presence of viable cells at both time points in all the conditions (Fig. 2a). At 54 days culture, all samples conditions did not show any statistically significant decrease in cell viability when compared to *T*_0_ samples. No statistically significant differences in cell viability were also observed between the two different types of mesh implants (Fig. 2b). This staining technique also revealed the appearance of elongated viable fibroblast-like cells within all sample at 54 days.

### Tissue morphology

**Fig. 3:**
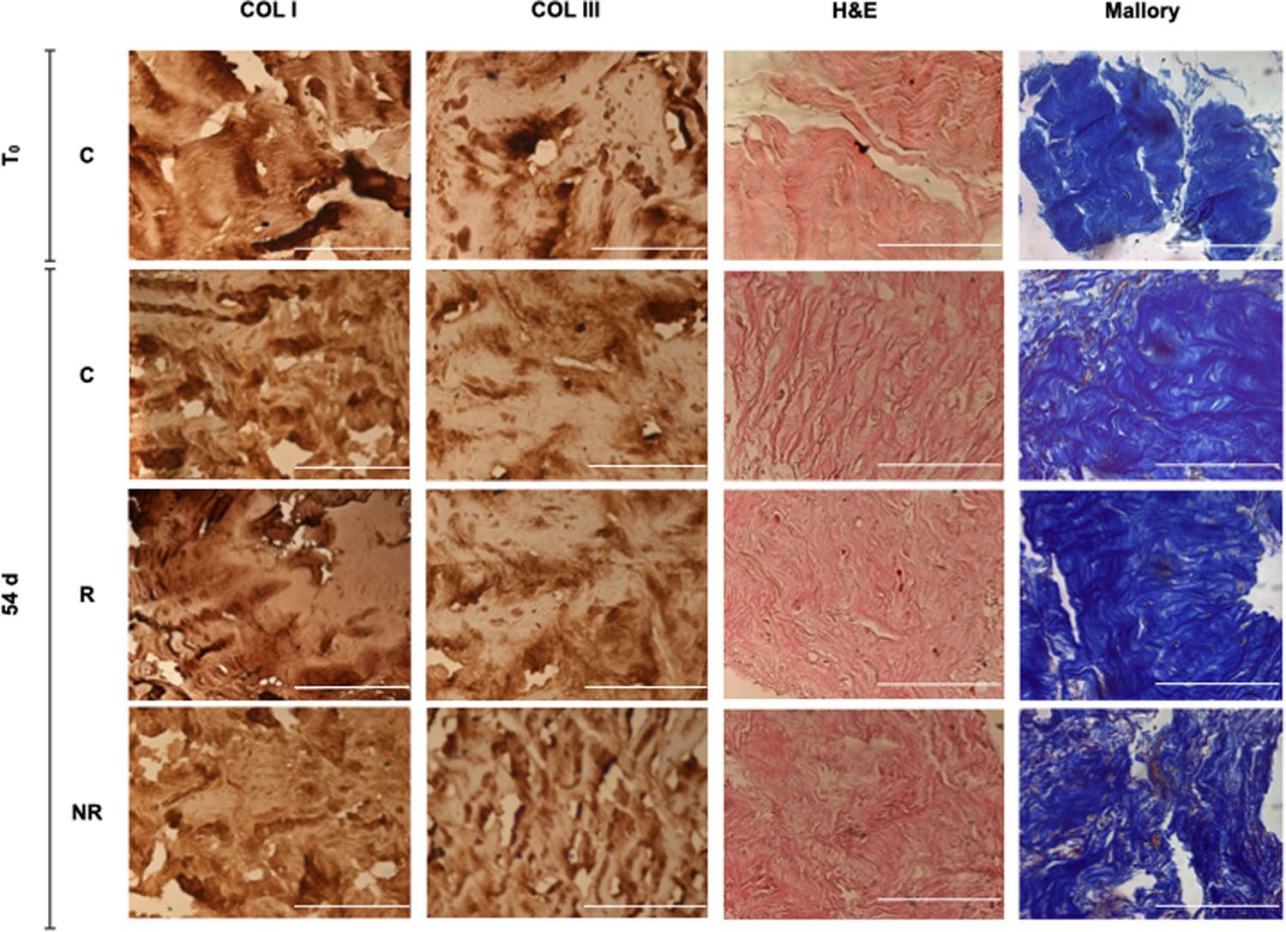
Representative immunohistochemistry (IHC), Hematoxylin and Eosin (H&E) and Mallory trichrome staining images (40X magnification, scale bar = 100 µm). *C* samples which did not undergo mesh implant, mesh-free controls, *R* fully resorbable poly-4-hydroxy-butyrate mesh implant, *NR* non-resorbable polypropylene mesh implant, *COL I* collagen I staining, *COL III* collagen III, *T*_*0*_ time of tissue samples arrival, *54d* 54 days culture from mesh implantation

Both Mallory trichrome staining and Hematoxylin and Eosin staining revealed a diffuse fibrous appearance in all sample conditions, with collagen representing the majority of the microscopic field (Fig. 3).

Interestingly, samples which underwent mesh implants placement showed an architectural collagen pattern distortion with tangled and twisted fibers orientation when compared to mesh-free samples wavy parallel bundles (Fig. 3).

Moreover, samples showed minor cellular component within collagen fibers, consisting mainly of elongated fibroblastoid cells. Muscle fibers presence, visible as red staining using Mallory trichrome, was rare and represented just a minimal portion of the samples (Fig. 3).

### Type I/III collagen immunostaining

Positive immunostaining for type I collagen appeared thick and heavy, with a widely distributed pattern in all samples (Fig. 3). Conversely, type III collagen immunostaining was lighter, sparser and unevenly distributed.

The semi-quantitative analysis of collagen immunostaining (relative to both collagen I and collagen III) did not show any statistically significant difference between control mesh-free samples (C) at the two time points: *T*_0_ and 54 days (data relative to the individual samples are shown in Table 2).

**Table 2:** Fascia samples Type I and Type II collagen immunostaining quantification at the two different time points on individual subject level

Time-point	Condition	Collagen type	GB 001	GB 002	GB 003	GB 004	GB 005	GB 006	GB 007	MEAN	*P*-value		
*T*_0_	C	I	0.34 ± 0.084	0.47 ± 0.066	0.51 ± 0.065	0.44 ± 0.055	0.39 ± 0.017	0.40 ± 0.016	0.32 ± 0.027	0.41 ± 0.03	NS vs C 54d		
		III	0.37 ± 0.026	0.45 ± 0.029	0.42 ± 0.042	0.39 ± 0.009	0.32 ± 0.010	0.32 ± 0.004	0.19 ± 0.017	0,35 ± 0.03	NS vs C 54d		
54d	C	I	0.41 ± 0.043	0.34 ± 0.031	0.28 ± 0.007	0.27 ± 0.020	0.34 ± 0.013	0.49 ± 0.009	0.41 ± 0.027	0.36 ± 0.03	NS vs C *T*_0_	0.025 vs R	NS vs NR
		III	0.39 ± 0.041	0.37 ± 0.067	0.33 ± 0.026	0.33 ± 0.009	0.32 ± 0.012	0.49 ± 0.003	0.44 ± 0.009	0.38 ± 0.02	NS vs C T_0_	0.015 vs R	NS vs NR
	R	I	0.43 ± 0.060	0.43 ± 0.061	0.32 ± 0.014	0.34 ± 0.031	0.50 ± 0.023	0.50 ± 0.003	0.47 ± 0.040	0.43 ± 0.03	0.025 vs C 54d	0.023 vs NR	
		III	0.31 ± 0.033	0.34 ± 0.013	0.26 ± 0.002	0.27 ± 0.015	0.31 ± 0.012	0.37 ± 0.018	0.33 ± 0.032	0.31 ± 0.01	0.015 vs C 54d	NS vs NR	
	NR	I	0.41 ± 0.045	0.40 ± 0.026	0.32 ± 0.057	0.32 ± 0.010	0.35 ± 0.026	0.39 ± 0.007	0.42 ± 0.023	0.37 ± 0.02	NS vs C 54d	0.023 vs R	
		III	0.37 ± 0.010	0.35 ± 0.037	0.30 ± 0.027	0.29 ± 0.007	0.30 ± 0.003	0.37 ± 0.010	0.43 ± 0.052	0.34 ± 0.02	NS vs C 54d	NS vs R	

Data are reported as mean optical density (OD) ± SEM of five randomly selected 40X whole-stained microscopic fields for each sample condition

Statistical comparisons between multiple groups were performed using RA test with Tukey’s post-hoc test

*C* samples which did not undergo mesh implant, mesh-free controls, *R* fully resorbable poly-4-hydroxy-butyrate mesh implant, *NR* non-resorbable polypropylene mesh implant, *T*_*0*_ time of tissue samples arrival, *54d* 54 days culture from meshes implantation, *NS* not statistically significant

Differences with *P* < 0.05 were considered statistically significant

**Fig. 4:**
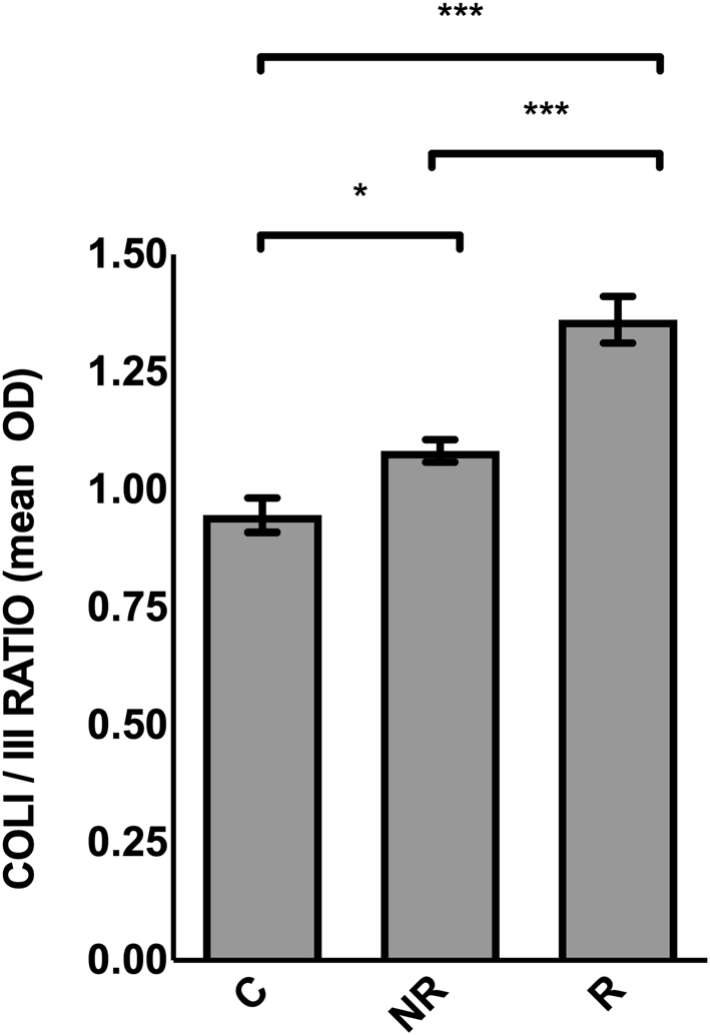
Collagen I/III immunostaining ratio in different samples conditions after 54 days of culture. Results are expressed as optical density (OD). Data are reported as mean ± SEM (*n* = 7 subjects). For each subject, the mean OD of five randomly selected 40X whole-stained microscopic fields for each sample condition was calculated. Statistical comparisons between multiple groups were performed using RA test with Tukey’s post-hoc test. Differences with *P* < 0.05 were considered significant. *C* samples which did not undergo mesh implant, mesh-free controls, *R* fully resorbable poly-4-hydroxy-butyrate mesh implant, *NR* non-resorbable polypropylene mesh implant, *T*_*0*_ time of tissue samples arrival, *54d* 54 days culture from meshes implantation; *** = *P* < 0.001; * = *P* < 0.05

On the other hand, the semi-quantitative analysis on samples cultured ex vivo for 54 days after meshes fixation, showed a slight (1.08 ± 0.02 vs. 0.95 ± 0.04), although statistically significant (*P* = 0.027), increase in the collagen I/III ratio in samples implanted with non resorbable meshes (NR) compared to samples in control culture conditions (C, i.e. no added meshes). A more evident (1.36 ± 0.05 vs. 0.95 ± 0.04), highly statistically significant, increase in the collagen I/III ratio was observed in samples implanted with resorbable meshes (R), compared to C (*P* < 0.001). A statistically significant difference between R and NR conditions (1.36 ± 0.05 vs. 1.08 ± 0.02, *P* < 0.001) also emerged (Fig. 4). In particular, as evident in Table 2, while collagen I staining intensity was significantly higher in the R group compared to both C and NR groups, the staining intensity of collagen III was significantly lower in the R group compared to controls (C).

## Discussion

In the present study we provide for the first time evidence of the suitability of a novel ex vivo tissue model of abdominal hernia mesh implant to determine the effects of two different mesh implant materials (resorbable poly-4-hydroxybutyrate and non-resorbable polypropylene) on collagen production and fascial tissue remodeling in vitro. First evidence was obtained that the in vitro culture of human fascial tissues with the fully-resorbable mesh implants increases the collagen I/III ratio towards higher values compared to non-resorbable ones, which suggests a better performance of resorbable meshes in tissue remodeling.

As expected from previously published in vivo data [17], both resorbable and non-resorbable meshes appeared intact and with no signs of reabsorption after 54 days from implantation.

Evaluation of fascia samples tissue morphology revealed an organized parallel wavy collagen fibers pattern in mesh-free samples conditions at both time points, while a more disorganized and tangled orientation appeared in both mesh implants conditions. This change is representative of fascial scar tissue formation which plays a key role in surgical implant-induced abdominal wall reinforcement, along with neovascularization and deposition of extracellular matrix.

The developed culture method showed no significant impact on collagen content modification, as confirmed by immunostaining quantification results in mesh-free samples at the two time intervals. This results also confirms the possibility to assess collagen content in fascia tissue specimens despite innate primary samples variability from donor to donor.

The absence of significant cell viability loss between the two time points (*T*_0_ and 54 days after meshes implantation) confirms the short-term biocompatibility of both mesh materials. This also indicates the feasibility of the tridimensional culture method developed for this study, possibly allowing its establishment as a novel ex vivo model of surgical soft tissue repair.

Moreover, the appearance of viable elongated cells within the samples at 54 days may represent the presence of proliferating fibroblasts which usually occurs during the first stages of wound healing and allows collagen deposition at the site of mesh implantation.

However, it has to be noticed that collagen deposition, fibrosis and tissue remodeling are usually evaluated at longer mid-term time intervals in vivo (12–24 weeks from implantation) and any ex-vivo model obviously lacks in several biological mechanisms which could affect collagen deposition and foreign body response in a full organism.

The higher collagen I/III ratio observed in the resorbable mesh group when compared with both mesh-free samples and the non-resorbable mesh group, may be likely caused by differences in implant materials biocompatibility. The higher cell affinity of butyrate has already been demonstrated specifically compared to polypropylene [18–21]. Moreover, surgical meshes colonization and subsequent collagen I deposition by fibroblasts is a well-known process which has been observed both in vivo and in vitro [22, 23]. In this scenario, resorbable P4HB meshes might allow fibroblasts colonization, their proliferation, activation and the ensuing deposition of mature collagen once in contact with the fascial tissue, at higher levels compared to polypropylene-containing meshes. This could eventually lead to the observed increase in collagen I/III ratio, which is representative of functional fascial tissue repair. Overall, the results we obtained confirm previously published short- and mid-term in vivo findings suggesting a more favorable long-term tissue remodeling outcome obtainable with resorbable P4HB mesh implants [21, 24, 25].

Finally, considering the key role of collagen I/III ratio in influencing connective tissue biomechanical functionality and its reported decrease in fascia specimens from patients with abdominal hernia, our results suggest how fully resorbable P4HB mesh implants could allow for higher quality tissue repair and functionality restoration if compared to monofilament polypropylene.

In conclusion, the present study offers valuable preliminary results about surgical meshes-induced tissue remodeling in fascia samples and highlights the feasibility and promising role of the proposed ex vivo model.

The results we obtained definitely warrant further validation in a larger and independent sample cohort, which also includes fascia samples from patients with abdominal hernia and hernia-prone subjects (such as obese, immunodepressed and diabetic patients). This will contribute to further confirm the utility of the proposed model in studying tissue remodeling in surgical hernia repair.

In addition, it will be possible to apply the ex vivo model we developed in the present study, to mechanotransduction studies, applying exogenous mechanical forces together with different meshes implantation. This will eventually allow for a more complex biomechanical characterization of mesh-induced soft tissue repair, potentially validating the use of the novel ex vivo model we developed to predict whether a certain tissue specimen from patients will remodel differently, depending on the type of mesh material.
